# In Vitro Validation of the Therapeutic Potential of Dendrimer-Based Nanoformulations against Tumor Stem Cells

**DOI:** 10.3390/ijms23105691

**Published:** 2022-05-19

**Authors:** Nadezhda Knauer, Valeria Arkhipova, Guanzhang Li, Michael Hewera, Ekaterina Pashkina, Phuong-Hien Nguyen, Maria Meschaninova, Vladimir Kozlov, Wei Zhang, Roland S. Croner, Anne-Marie Caminade, Jean-Pierre Majoral, Evgeny K. Apartsin, Ulf D. Kahlert

**Affiliations:** 1 Research Institute of Fundamental and Clinical Immunology, 630099 Novosibirsk, Russia; nuknauer@niikim.ru (N.K.); pashkina.e.a@yandex.ru (E.P.); vakoz40@yandex.ru (V.K.); 2 Clinic for Neurosurgery, Medical Faculty, Heinrich-Heine University Düsseldorf, 40225 Düsseldorf, Germany; michael.hewera@med.uni-duesseldorf.de; 3 Institute of Chemical Biology and Fundamental Medicine SB RAS, 630090 Novosibirsk, Russia; v.arkhipova@g.nsu.ru (V.A.); mesch@niboch.nsc.ru (M.M.); 4 Department of Natural Sciences, Novosibirsk State University, 630090 Novosibirsk, Russia; 5 Department of Molecular Neuropathology, Beijing Neurosurgical Institute, Capital Medical University, Beijing 100054, China; liguanzhang122@163.com; 6 Center of Molecular Medicine, Department I of Internal Medicine, University of Cologne, 50923 Cologne, Germany; hien.nguyen@uk-koeln.de; 7 Department of Neurosurgery, Beijing Tiantan Hospital, Capital Medical University, Beijing 100054, China; zhangwei_vincent@126.com; 8 China National Clinical Research Center for Neurological Diseases, Beijing 100053, China; 9 Chinese Glioma Genome Atlas Network (CGGA), Asian Glioma Genome Atlas Network (AGGA), Beijing 100070, China; anne-marie.caminade@lcc-toulouse.fr (A.-M.C.); jean-pierre.majoral@lcc-toulouse.fr (J.-P.M.); 10 Molecular and Experimental Surgery, Clinic for General-, Visceral-, Vascular-, and Transplant-Surgery, Medical Faculty, University Hospital Magdeburg, 39120 Magdeburg, Germany; roland.croner@med.ovgu.de; 11 Laboratoire de Chimie de Coordination CNRS, 31077 Toulouse, France

**Keywords:** Lyn, 3D tumor models, dendrimers, phosphorus, nucleic acid therapeutics, polyelectrolyte complexes, tumor stem cells, nanomedicine

## Abstract

Tumor cells with stem cell properties are considered to play major roles in promoting the development and malignant behavior of aggressive cancers. Therapeutic strategies that efficiently eradicate such tumor stem cells are of highest clinical need. Herein, we performed the validation of the polycationic phosphorus dendrimer-based approach for small interfering RNAs delivery in in vitro stem-like cells as models. As a therapeutic target, we chose Lyn, a member of the Src family kinases as an example of a prominent enzyme class widely discussed as a potent anti-cancer intervention point. Our selection is guided by our discovery that Lyn mRNA expression level in glioma, a class of brain tumors, possesses significant negative clinical predictive value, promoting its potential as a therapeutic target for future molecular-targeted treatments. We then showed that anti-Lyn siRNA, delivered into Lyn-expressing glioma cell model reduces the cell viability, a fact that was not observed in a cell model that lacks Lyn-expression. Furthermore, we have found that the dendrimer itself influences various parameters of the cells such as the expression of surface markers PD-L1, TIM-3 and CD47, targets for immune recognition and other biological processes suggested to be regulating glioblastoma cell invasion. Our findings prove the potential of dendrimer-based platforms for therapeutic applications, which might help to eradicate the population of cancer cells with augmented chemotherapy resistance. Moreover, the results further promote our functional stem cell technology as suitable component in early stage drug development.

## 1. Introduction

The development of adequate effective therapies against malignant tumors such as glioblastoma, the most aggressive type of brain cancer, represent a clinically unmet need. Tumor malignancy is characterized by elevated levels of therapy resistance and invasive growth patterns. It is believed that malignant features of gliomas and glioblastomas are promoted by cells with stem cell properties [1]. Therapeutics that target such cancer stem cells (CSCs) are of the highest clinical need. Ideally, such treatments should be able to (a) be efficiently directed towards a molecular target that plays essential roles to maintain the CSC pool, and (b), to be developed in high amounts, with limited costs meanwhile being highly standardized.

Oncogenic Lyn, a member of the prominent kinase family Src, is most precisely studied in the context of hemoblastosis such as lymphomas, and has previously been implemented as a potent regulator of the biology of brain cancer, however with hitherto conflicting observations revealing its anti-tumorigenic and pro-tumorigenic potential. In addition, most of the studies are based on preclinical data only, whereas clinical studies do not interrogate current state of the art tumor diagnostics and ignore ethnic diversity. In our project we aim to reassess the role of Lyn in glioma by benchmarking its function as a druggable therapeutic in three-dimensional disease models recapitulating the spectra of transcriptional subtypes of glioblastoma and different prominent brain cancer DNA mutation patterns [2]. Moreover, we apply current molecular neuropathology to associate Lyn activation with accepted clinical diagnostic marks.

Despite numerous studies that have appointed Lyn as a key molecule of tumorigenesis, specific inhibitors for this kinase are still lacking. Several inhibitors can potently block the kinase activity of Lyn as part of a pan-SFK inhibition through their interaction with the catalytic domain, but all have wide spectra of additional targets, most likely due to the homolog of the fold of the kinase domain, which is similar to all other known protein kinases [3,4]. This can partially explain the limited efficacy of dasatinib in some tumor entities including glioblastoma, despite its high potency to induce tumor cell death [5,6]. Thus, the specific and efficient suppression of Lyn expression and activity for therapeutic purposes remains to be invented.

An emerging field in precision medicine was facilitated with the discovery of RNA interference (RNAi) that efficiently downregulates gene expression. RNAi technology not only compensates for the lack of inhibitors of many molecules, but their high specificity and ability to overcome resistant mutations also gives this gene therapy a promising therapeutic solution for targeting molecules such as Lyn [7]. Since the need for RNAi delivery systems varies depending on the targeted tissue, this work is aimed to develop nanoformulations containing efficacy-validated anti-Lyn payloads (siLyn).

Over the last 15 years, dendrimers, a platform based on synthetic chemistry, emerged as a potent technology for nanomedicine. Dendrimers are highly symmetrical hyperbranched tree-like macromolecules, having a central core and branch units growing radially [6,8,9,10]. By choosing the structural elements of dendrimers, it is possible to design and to synthesize dendrimers with specific physicochemical properties and biological behaviors [7,11,12].

Cationic phosphorus dendrimers, i.e., dendrimers containing phosphorus atoms as core and branching points, show promise for the antitumor therapy as carriers for therapeutic oligonucleotides and also as bioactive entities both as agents for drug delivery [9,13,14,15,16,17,18,19] and as nanodrugs per se [20,21,22,23,24,25,26]. The former option is realized due to the presence of multiple cationic groups on the periphery of dendritic molecules that provide efficient oligonucleotide binding and protection against serum nucleases. The mechanisms of the latter one is not yet well elucidated and likely involves the selective induction of Bax-dependent apoptosis in target cells [27].

Herein, we report nanoformulations based on cationic phosphorus dendrimers designed either for Lyn knockdown by RNAi or for antitumor activity per se as well as present a biological evaluation of this platform on a collection of pathophysiological relevant in vitro disease models of brain tumor stem cells.

## 2. Materials and Methods

### 2.1. Disease Models

We used several glioma cell models including a classical glioblastoma culture (U87) and three stem cell models of glioblastoma (BTSC233, JHH520, NCH644). These models were kindly provided by colleagues: BTSC233 (M.S. Carro, Freiburg University, Freiburg im Breisgau, Germany); JHH520 (G. Riggins, Johns Hopkins, Baltimore, MD, USA); NCH644 (C. Herold-Mende, Heidelberg University, Heidelberg, Germany); U87 (A. Weyerbrock, Department General Neurosurgery, Medical Center Freiburg, Germany).

**Figure A1:**
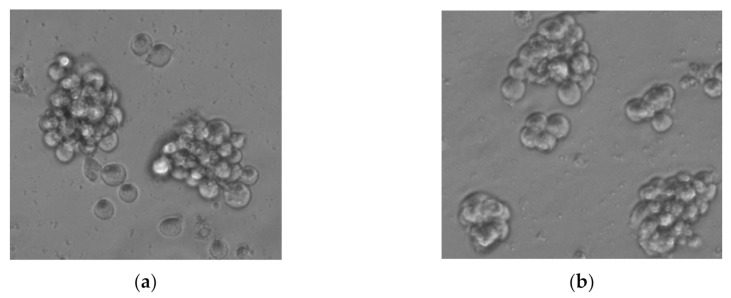
Microscopy images of neurospheres: JHH520 cells (**a**) and NCH644 cells (**b**). Magnification 200x.

All cells were cultivated as neurospheres (Figure A1, in Appendix A) in DMEM media (high-glucose, no pyruvate, Thermo Fisher, Waltham, MA, USA), containing F12 (3:1) and 1X B27 supplements (both Thermo Fisher, Waltham, MA, USA), 20 ng/mL human EGF, 20 ng/mL human VGF (both Peprotech, Hamburg, Germany), 5 µg/mL heparin (Sigma-Aldrich, Taufkirchen, Germany), 1X penicillin/streptomycine (Sigma-Aldrich, Taufkirchen, Germany) in standard conditions (humidified 37 °C, 5% CO_2_) [28]. For all assays, volume-adjusted medium was used as mock treatment. Ethical approval to conduct the in vitro studies was granted by the ethics commission of the Medical Faculty of the Heinrich-Heine University (study ID 5841R).

### 2.2. Patient Data Resources and In Silico Analysis

Molecular and clinical data of glioma patients were obtained from the publicly assessable sources, namely The Chinese Glioma Genome Atlas (CGGA, (https://www.cgga.org.cn, accessed on 2 October 2020; two independently acquired datasets of with n = 325 individuals, and 2019 dataset with n = 693 individuals) and The Cancer Genome Atlas (TCGA, https://tcgadata.nci.nih.gov, accessed on 2 October 2020; with n = 702 individuals, for verification). For the CGGA, molecular testing of each patient was performed at the Molecular Pathology Testing Center of the Beijing Neurosurgical Institute and the data acquisition was approved by the Beijing Tiantan Hospital institutional review board and tumor specimen quality control.

The statistical computations and figure drawing were performed with R package ‘ggplot2′. An unsupervised cluster analysis was performed by the *pheatmap* package in software environment R (Version 4.0.0). The analysis processes were carried out under the default parameters. A student’s *t*-test was used in the differential analysis of two groups in this study. The drawing was performed by the *ggplot2* package in software environment R (Version 4.0.0). The Kaplan–Meier survival analysis was used in the survival analysis of this study.

### 2.3. Functional Enrichment Analysis

Genes that were strongly correlated with Lyn expression were screened out by Pearson correlation analysis (the absolute value of R-value > 0.5 and *p*-value < 0.05). The above genes were uploaded to the Database for Annotation, Visualization and Integrated Discovery (DAVID) v6.8 for functional enrichment analysis. Homo sapiens was selected as the background for functional enrichment analysis. The visualization of functional enrichment analysis results was performed by EXCEL (Microsoft Office Professional Plus 2019). The functional enrichment analysis of Lyn in the three databases was also undertaken.

### 2.4. Therapeutic Formulations

**Figure 1:**
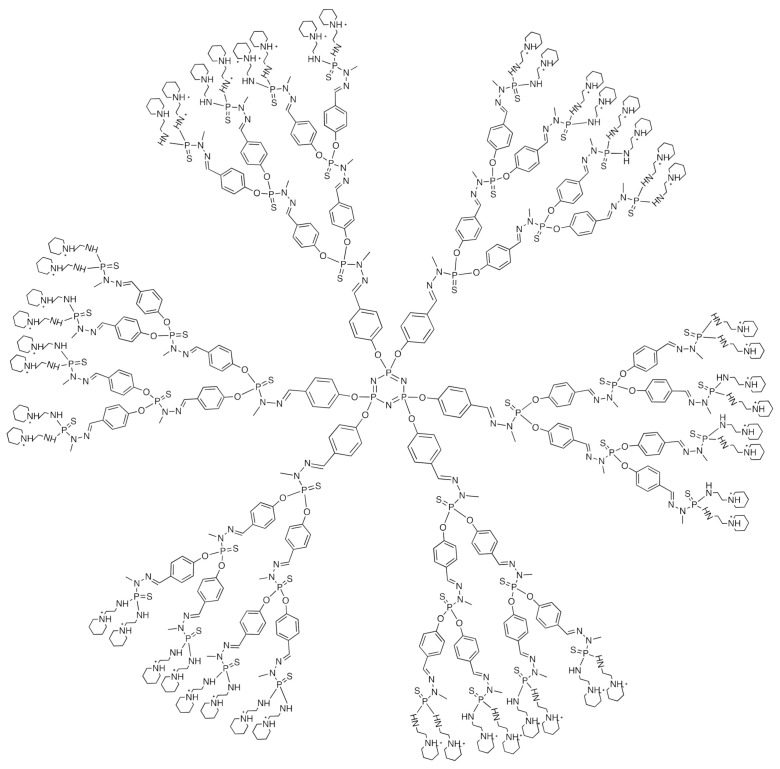
Chemical structure of cationic phosphorus dendrimer of the third generation (AE2G3).

The third-generation cationic phosphorus dendrimer AE2G3 was used (See Figure 1). The dendrimer was synthesized as previously described [9]. As therapeutic oligonucleotides, a set of four ON-TARGETplus Human Lyn siRNAs (Dharmacon, Lafayette, CO, USA) were used. We also used siGLO RISC-Free control oligonucleotide (Dharmacon, Lafayette, CO, USA) as a fluorescent-labelled RNA for the internalization control and a set of four scramble oligonucleotide duplexes [29]. The sequences are given in the SI. As low-molecular therapeutic entities, the anti-glioblastoma drugs temozolomide (Sigma-Aldrich, Taufkirchen, Germany) and Dasatinib (Selleckchem, Zürich, Switzerland) were used. Lipofectamine 3000 (Invitrogen, Waltham, MA, USA) was used as a standard for assessing siRNA transfection efficiency.

To prepare the dendrimer solution, its powder was dissolved in milli-Q water to the concentration 1 mM, and the solution was stored at 4 °C. Oligonucleotides were diluted in 1X siRNA buffer (Dharmacon, Lafayette, CO, USA) to a concentration of 20 mM, then stored at −20 °C. Temozolomide and Dasatinib were diluted in DMSO (dimethyl sulfoxide, Sigma-Aldrich, Taufkirchen, Germany) to the concentration 20 mM, then stored at −20 °C.

To prepare the dendriplexes solutions, we mixed the oligonucleotide solution and dendrimer solution in appropriate concentrations to reach the concentration 10 nM, 30 nM, 60 nM, 100 nM in RNA equivalent and 10-fold excess of cationic groups [9] for the cell treatment (charge ratio, CR = 10). Complexes were incubated for 15 min at room temperature (in dark for the fluorescently labelled molecules). Lipofectamine 3000/siRNA complexes were prepared according to the distributor’s protocol (Invitrogen, Waltham, MA, USA).

The sizes of siRNA-dendrimer complexes were measured by dynamic light scattering using the Zetasizer Nano-ZS (Malvern Instruments Ltd., Malvern, UK). Samples of 100 nM siRNAs were prepared in 1X siRNA buffer, then dendrimer was added to obtain given values of the cation:anion ratio (CR) in the samples from 0 to 10. Samples were incubated for 15 min at room temperature, and measurements were made in ZEN0040 cells.

### 2.5. Gel Electrophoresis

To characterize whether the complexation of siRNA with dendrimer protects it from RNAse-mediated degradation, we performed an agarose gel electrophoresis analysis [15]. AE2G3 was complexed with siLyn (100 nM in RNA equivalent), and free RNA was used as a control. RNAse A (VWR) treatment (2.5 μg/mL) was performed for 30 min in 37 °C, then samples were incubated on ice for 10 min. Heparin (Sigma-Aldrich, Taufkirchen, Germany) treatment (0.041 mg/mL) was used to displace RNA from the dendriplexes: it was added to samples and then incubated on ice for 10 min.

We evaluated the bands’ electrophoretic mobility in 3% agarose (Sigma-Aldrich, Taufkirchen, Germany) gel stained by SYBR Safe Gel Stain (Thermo Fisher, Waltham, MA, USA) for 45 min at 35 mA. GeneRuler 1 kb Plus DNA Ladder (Thermo Fischer, Waltham, MA, USA) was used as a marker of fragment length.

### 2.6. MTT Assay

Cells (BTSC233, JHH520, NCH644, U87) were seeded at 96-well flat-bottomed culture plates (10,000 cells in 100 µL total volume), treated by free drugs (AE2G3 or temozolomide; 0 µM; 0.1 µM; 1 µM; 10 µM; 100 µM) or complexes (AE2G3/siLyn, or Lipofectamin 3000/siLyn; 10 nM; 30 nM; 60 nM; 100 nM in RNA equivalent according to the charge ratio; or individual components in appropriate concentrations), then incubated for 72 h in standard conditions (humidified 37 °C, 5% CO_2_). For comparing the effect of dasatinib and siLyn-dendriplexes, we used dasatinib at the highest concentration corresponding to the maximum concentration of RNA—100 nM. Non-treated cells were used as a control (non-treated control, NTC), and complete medium was used as a blank control. Every point was made in five technical repetitions.

For performing MTT assay 10 µL MTT reagent (thiazolyl blue tetrazolium bromide, Sigma Aldrich) was added to every well and mixed thoroughly, and plates were incubated ca. 3 h in the dark at RT. Next, 100 µL MTT of Lysis buffer containing Isopropanol (VWR, Langenfeld, Germany), Triton X (Sigma Aldrich, Taufkirchen, Germany) and HCl (Roth, Karlsruhe, Germany) was added, thoroughly mixed, and incubated 20 min in the dark at RT. Absorbances at 570 nm and 650 nm were read on a Paradigm plate reader (Molecular Devices, San Jose, CA, USA).

### 2.7. Internalization of siRNA-Containing Complexes

Cells (BTSC233, JHH520) were cultivated in 12-well flat-bottom plates (100,000 cells in 500 µL total volume) in the presence of AE2G3/siGlo or Lipofectamine 3000/siGlo complexes (100 nM in RNA equivalent according to the charge ratio) or individual components in appropriate concentrations. Non-treated cells were used as a control (non-treated control, NTC). Cells were incubated in standard conditions (humidified 37 °C, 5% CO_2_) for 3 h then collected, washed twice by cold PBS (Gibco) with 10% FBS (fetal bovine serum, Gibco) and 2 mM EDTA (ethylenediaminetetraacetic acid, Sigma-Aldrich, Taufkirchen, Germany), then fixed by 4% PFA solution (paraformaldehyde, Sigma-Aldrich, Taufkirchen, Germany) and analyzed. To wash out molecules from the surface, treating by acidic glycine buffer (50 mM, pH 3.0) was performed after first PBS washing. Flow cytometry analysis was done on a CyAn (Daco, Beckman Coulter, Denver, CO, USA) machine using Summit (Beckman Coulter, Denver, CO, USA) software. Experiments were performed using three independent biological repetitions before statistical analysis.

### 2.8. Expression of Tumor Cells Surface Markers

Cells (BTSC233, JHH520, NCH644) were cultivated in 12-well flat-bottom plates (500,000 cells in 2 mL total volume) in the presence of 1 µM drug studied (AE2G3, temozolomide, dasatinib) in standard conditions for 48 h. Non-treated cells were used as a control (non-treated control, NTC). Cells were then collected and washed twice by cold PBS (Gibco) containing 10% FBS (fetal bovine serum, Gibco) and 2 mM EDTA (ethylenediaminetetraacetic acid, Sigma-Aldrich, Taufkirchen, Germany). For evaluating the expression of tumor cells’ surface markers, we used monoclonal antibodies conjugated with fluorochromes: TIM-3-FITC (Biolegend, Amsterdam, the Netherlands), PD-L1-PE (Biolegend, Amsterdam, the Netherlands), and CD47-APC (BD, Eysins, Switzerland). To distinguish dead and live cells, Annexin V-Pacific Blue antibodies (Biolegend, Amsterdam, the Netherlands) were used. Flow cytometry analysis was done on a CyAn (Daco, Beckman Coulter, Denver, CO, USA) cell analyser using Summit (Beckman Coulter, Denver, CO, USA) and Flow Jo (BD, Eysins, Switzerland) software. Experiments were performed using three independent biological repetitions before statistical analysis.

### 2.9. IL-10 Secretion

Supernatants collected after JHH520 and NCH644 cells treated by AE2G3, temozolomide (3 μM, 72 h) and AE2G3/siGlo or Lipofectamine 3000/siGlo complexes (100 nM in RNA equivalent according to the charge ratio, 48 h) or individual components in appropriate concentrations, were used to perform ELISA analysis by applying the Human IL-10 ELISA MAX Deluxe kit (Biolegend, San Diego, CA, USA) in accordance with distributor recommendations. Experiments were performed using three independent biological repetitions before statistical analysis. IL-10 expression values were expressed as a relative value to non-treated control (NTC).

### 2.10. Statistical Analysis

We used STATISTICA for Windows 10 (StatSoft) and GraphPad Prism 7 (GraphPad Software, San Diego, CA, USA) software for statistical analysis and data visualization.

The violin plots and Kaplan-Meier Curves were drawn by R language (v4.0). The significance of the difference between the two groups of patients was verified by unpaired *t*-test. The significance of the overall survival between the two groups of patients was verified by the Log Rank Test. A *p*-value < 0.05 is considered statistically significant.

## 3. Results

### 3.1. Dendrimer Alone Exhibits a Dose-Dependent Cytotoxic Activity towards Tumor Stem Cells

**Figure 2:**
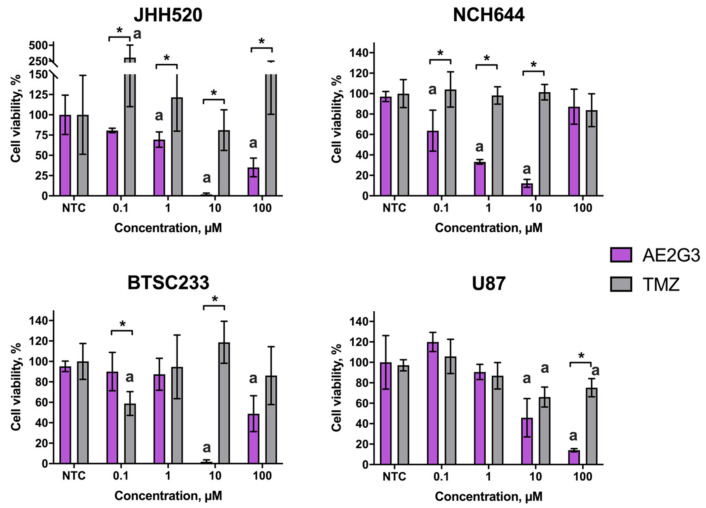
Evaluation of viability of cells of different glioblastoma cell lines (GSCs—JHH520, NCH644, BTSC233 and non-GSCs—U87) after 72 h of treatment by AE2G3 in comparison with standard-care treatment by temozolomide (TMZ). Asterisk (*) marks the significant difference (*p* < 0.05) between columns, letter “a” marks significant difference in comparison with non-treated control (NTC).

In general, treatment by AE2G3 could be characterized as having dose-dependent toxicity. In low concentrations, AE2G3 is less toxic for BTSC233 cells (0.1 µM, *p* = 0.02), but in higher doses it shows a higher effect (10 µM, *p* = 0.012) in comparison with TMZ (Figure 2).

JHH520 cells demonstrated decreased viability after AE2G3 treatment compared to TMZ treatment in the whole spectrum of concentrations (*p* = 0.03; 0.02; 0.02 and 0.12 respectively). A similar effect was observed for NCH644 cells (0.1 µM, *p* = 0.037; 1 µM, *p* = 0.03; 10 µM, *p* = 0.02) and SF188 cells (1 µM, *p* = 0.02; 10 µM, *p* = 0.007). For U87 cells this effect was observed only for the highest concentration of 100 µM (*p* = 0.03). AE2G3 in the lowest dose 0.1 µM was less toxic for SF188 cells (*p* = 0.02).

Comparing different types of glioblastoma cell cultures, we found that U87 cells are more robust to AE2G3 treatment in low and medium concentrations than BTSC233 cells (10 µM, *p* = 0.12), JHH520 cells (0.1 µM, *p* = 0.03; 1 µM, *p* = 0.037; 10 µM, *p* = 0.02), NCH644 cells (0.1 µM, *p* = 0.03; 1 µM, *p* = 0.03; 10 µM, *p* = 0.012) and SF188 (0.1 µM, *p* = 0.02; 1 µM, *p* = 0.03; 10 µM, *p* = 0.007). In high dose (100 µM) U87 cells have lower or similar viability in comparison to other cell types (*p* = 0.02 for BTSC233; *p* = 0.02 for JHH520; *p* = 0.03 for NCH644 and *p* = 0.052 for SF188).

### 3.2. Free AE2G3 Changes the Expression of Surface Markers Characterizing Interaction of Tumor Cells with Immune Microenvironment (TIM-3, PD-L1, CD47)

**Figure 3:**
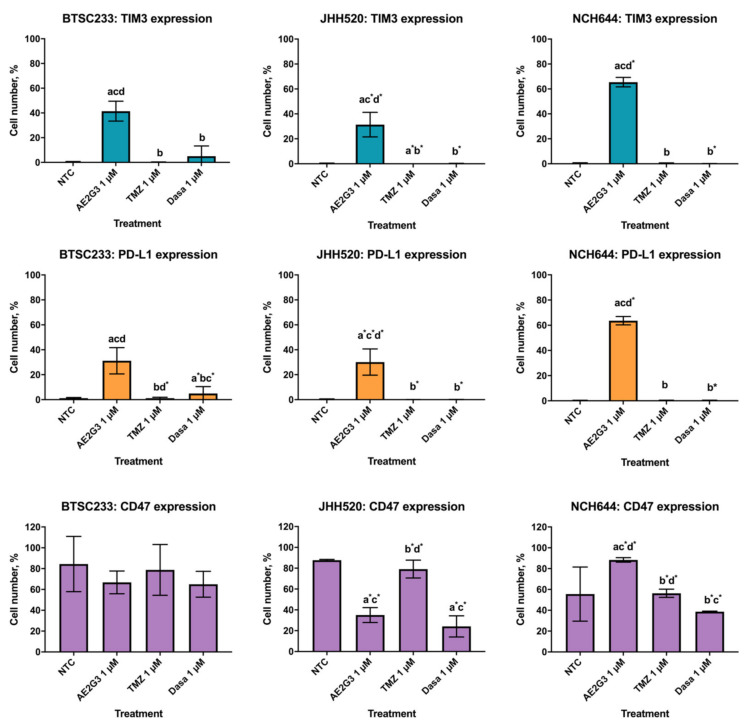
Evaluation of expression of surface markers (TIM3, PD-L1, CD47) on cells of GSCs cell cultures after treatment of cationic phosphorus dendrimer AE2G3 in comparison with non-treated control (NTC), temozolomide (TMZ) and dasatinib (Dasa). “a” marks significant difference in comparison with NTC, “b” marks significant difference from AE2G3 treatment, “c” marks significant difference with TMZ treatment and the letter “d” marks a significant difference in comparison with Dasa (*p* < 0.05). Letters with an asterisk * mark the tendency (0.08 < *p* < 0.05).

We found that treatment by AE2G3 significantly increased the expression of TIM3 and PD-L1 in all cell cultures studied in comparison with non-treated controls. Interestingly, that effect was higher than for temozolomide and dasatinib (Figure 3). At the same time, AE2G3 did not change CD47 expression in BTSC233 cells, increased it in NCH644 cells, but decreased it in JHH520 cells. Neither chemodrug changed this parameter significantly.

### 3.3. Elevated Lyn Expression Predicts for Shorter Overall Survival Time of Glioma Patients and Associates to Clinical Negative Diagnostic Markers

**Figure A2:**
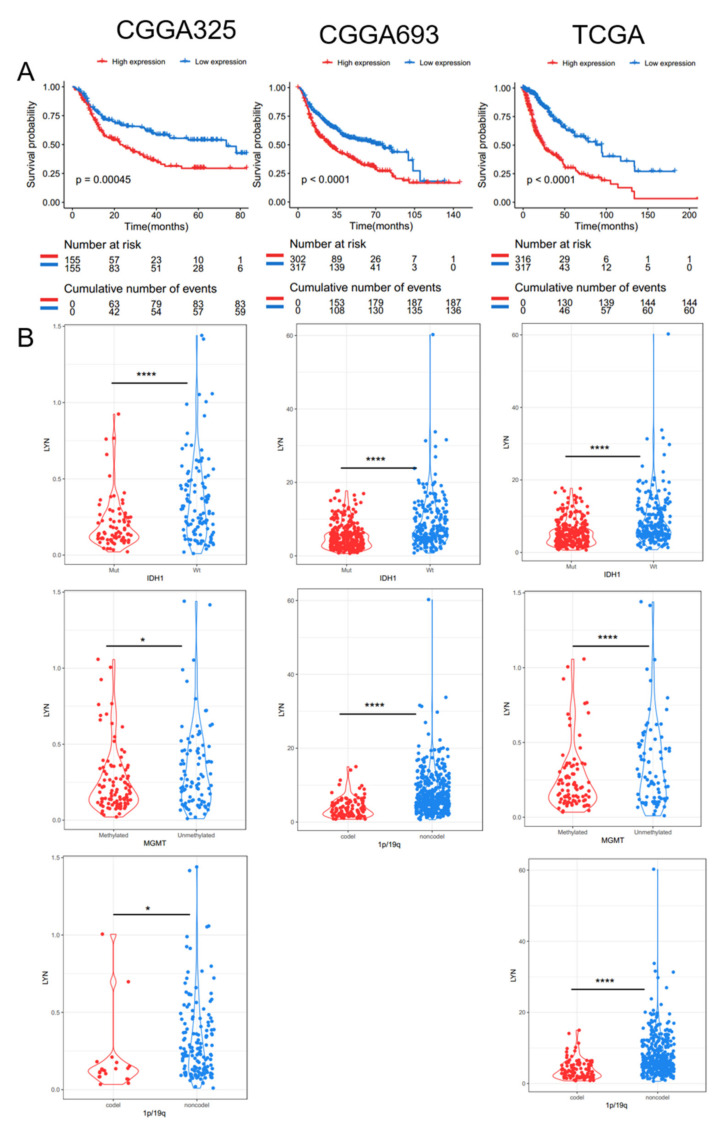
Data assessment in ethnic and gender diverse, independent molecular datasets of clinical glioma samples (CGGA325, CGGA693, TCGA). Elevated mRNA expression in glioma tissue predicts a significant shorter overall survival time of patients in all datasets (**A**). Moreover, Lyn transcripts are significantly enriched in tumors carrying IDH1 wildtype DNA, the unmethylated MGMT promoter, and DNA non-deleted for chromosome arms 1p/19q ((**B**), data on MGMT status in CGGA693 not available). * *p* < 0.05; **** *p* < 0.001.

We present the landscape of transcription of Lyn mRNA and their relationship between diverse diagnostic relevant, clinicopathological factors in two independent CGGA databases as well as in the TCGA dataset. Elevated Lyn expression levels are significant negative predictors for overall survival time of the patients (Figure A2A, in Appendix A). To the best of our knowledge, this is the ethnically most diverse and largest number of cancer patient samples probed for clinical prognostic values of Lyn. Given these strong indications, we then interrogated data of state-of-the-art molecular characterization of brain tumors to dissect any relevance of Lyn as a potential molecular- subtype specific therapeutic target. Interestingly, elevated Lyn transcription significantly coexists in tumors carrying IDH1 wildtype DNA, DNA with unmethylated MGMGT promoter and tumors that do not carry 1p/19q chromosome arm deletion (Figure A2B, in Appendix A). This data strongly associated with high Lyn expression may indicate glioblastoma tumorigenesis, as the mentioned molecular factors are defining neuropathological diagnostic parameters for glioblastoma according to current WHO guidelines [30].

### 3.4. AE2G3 Dendrimer Binds siLyn into Polyelectrolyte Complexes

**Figure 4:**
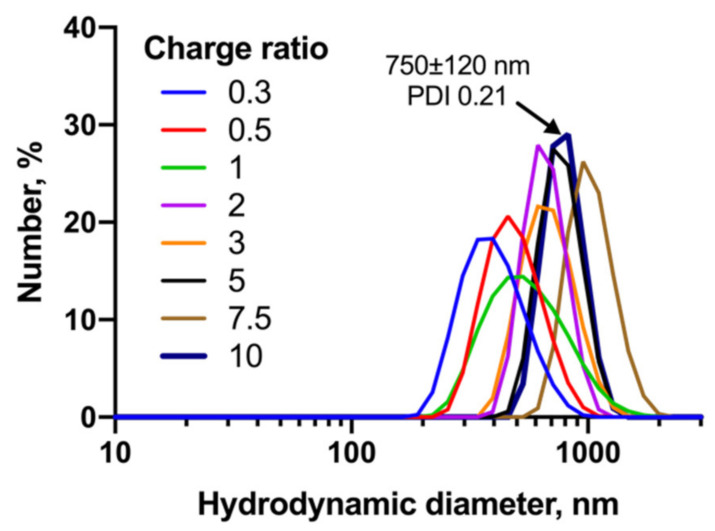
Dynamic light scattering profiles of AE2G3/siLyn dendriplexes obtained at different charge ratio (indicated on graph). Bold line represents the ratio used for further work, and the parameters of this complex are given on the graph.

AE2G3 efficiently binds siLyn to form dendriplexes (Figure 4). The size and polydispersity of complexes depends on the dendrimer:siRNA charge ratio (i.e., the excess of cations over anions). To obtain complexes possessing cationic surface charge to facilitate internalization, we have chosen a charge ratio of 10 for further experiments.

We found that the complexation with the AE2G3 dendrimer efficiently protects siRNA from digestion with RNAse A, the major nuclease of human blood (Figure S1). Therefore, we consider dendriplexes resistant to the biological media. Interestingly, heparin, a common polyanionic glycosaminoglycan in human blood, did not displace oligonucleotides from the complex.

### 3.5. Dendriplexes Are Efficiently Internalized into Tumor Cells

**Figure 5:**
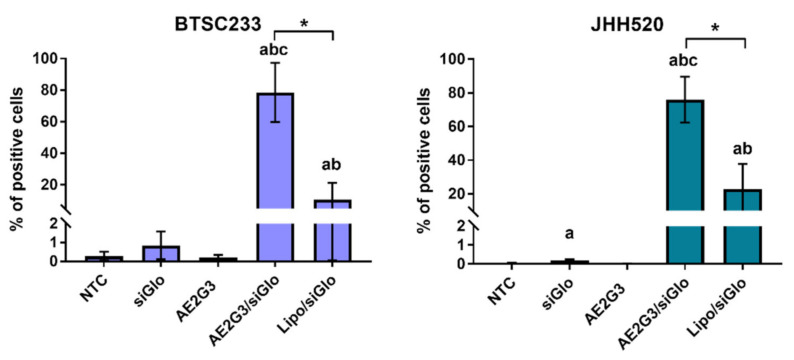
Evaluation of internalization of AE2G3 complexes with fluorescent-labelled RNA (siGlo) into glioblastoma stem-like cells BTSC233 and JHH 520 after 4 h of treatment in comparison with Lipofectamine 3000 (Lipo) complexes. Letters mark significant differences (*p* < 0.05): “a”—vs. NTC, “b”—vs. siGlo, “c”—in vs. free AE2G3. Bars with asterisks (*) show significant differences between two variants of complexes.

We found that siGlo oligonucleotide alone could be internalized (Figure 5) into tumor cells (*p* = 0.0143). Complexation with AE2G3 seems to be more effective for siRNA internalization than the use of Lipofectamine 3000 (*p* = 0.0143 for both cell cultures).

### 3.6. Dendrimer/siRNA Dendriplexes Decrease the Viability of Lyn-Expressing Tumor Stem-Cells

After comparison of sensitivity of different cell lines to treatment, we found that the viability of NCH644 cells is higher than JHH520 cells after treatment by dasatinib (*p* = 0.03) and siLyn–30 nM (*p* = 0.022), 60 nM (*p* = 0.02) and 100 nM (0.012). AE2G3 in the lowest concentration (80 nM) demonstrated higher toxicity on JHH520 than on NCH644 cells (*p* = 0.03), but for higher concentrations no significant differences were found. Interestingly, JHH520 cells were more robust to treatment by siLyn-containing complexes than NCH644 cells (*p* = 0.04 for AE2G3/siLyn 80/10 nM; *p* = 0.02 for Lypofectamine 3000/siLyn complexes with siLyn concentration of 30 nM and 60 nM).

## 4. Discussion

Glioblastoma cancer stem cells (GSCs) are a self-renewal tumor cell subset related with tumor initiation, progression and relapse [31,32,33,34] that makes GSCs a prospective target for glioblastoma therapy. However, GSCs are known to exhibit resistance towards temozolomide treatment, a standard care for glioblastoma. GSCs were shown to express multidrug resistance-related proteins (MGMT) [3,31,32,34,35,36] and to produce extracellular vesicles, which are disseminating factors of therapy resistance [31]. Furthermore, standard treatment with temozolomide promotes drug-induced tumorigenicity, i.e., de-differentiation of non-GSCs tumor cells into GSCs [5,33,34]. Considering the poor clinical prognosis for glioblastoma along with the resistance of GSCs to standard therapy, the development of new molecular-targeted approaches to effectively eradicate GSC is of the highest clinical need.

Targeting the activation of Src kinases is a promising and potent therapeutic strategy in oncology. The sector prominently features the clinically approved oncology blockbuster dasatinib (compound patent Sprycel with annual turnover of over USD 1 billion, www.cohausz-florack.de, accessed on 25 January 2022) and clinical assessment of various other inhibitors of this drug class underway [37]. However, pharmacological inhibitors that block one of the selected 11 sister members of the Src family, thereby potentially reducing off-target effects observed with pan-Src inhibitors while increasing the blockage efficacy of the predominantly tumorgenic Src member in the respective disease class, are missing. Stettner et al. reported that LYN is the most predominant active member of the Src family members in glioma [38]. Subsequent studies in glioma revealed both the pro-tumorigenic potential of Lyn, featuring its mediation of augmented stress resistance of gliomas [39], invasive potential [40], EGFR downstream mediator to promote cellular migration [41], as well as a tumor-suppressive role [42]. Moreover, in silico analyses identified Lyn as a core component defining the glioblastoma specific protein-protein interaction network [43]. Given this conflicting data, and the fact that hitherto only one publication probing Lyn activation in large scale clinical samples using American dataset REMBRANDT [40], our data fills a lack of knowledge and promotes Lyn to serve a clinical relevant therapeutic target in glioma. Since current WHO guidelines define the wildtype IDH1 locus as a defining mark to diagnose the neoplasm as glioblastoma [30], our data supports elevated Lyn mRNA abundancy as a compliment marker indicative for glioblastoma. Interestingly, we find Lyn elevated in tumors that are putatively less resistant to standard chemotherapy TMZ as designated by the methylated promoter of methyltransferase MGMT.

Altogether, since hitherto clinical trials using pan-Scr inhibitors as mono- or in the context of combination therapy are insufficiently successful [44,45,46], this data suggests that developing a Lyn-specific modulator of activation may possess biological and clinical relevance. In this point our results correlate with the data published recently [47]. The downregulation of Lyn in glioblastoma might reduce the cell proliferation ability and cell viability that can have important implications for anti-glioblastoma therapy. We aimed to functionally assess the appropriateness of Lyn as a target for siRNA therapy upon dendrimer-based delivery.

A common way to achieve selective silencing of a target gene is to use siRNA-based treatment. In particular, Lyn-specific siRNAs (siLyn) have already been described, and their efficiency validated in vitro [29]. However, nucleic acids have poor penetration ability into cells, therefore siRNA-based therapy needs carriers providing cell internalization and protecting siRNAs from degradation [9,48]. As mentioned above, cationic phosphorus dendrimers can serve as robust tools for this purpose.

Herein, we used the cationic phosphorus dendrimer AE2G3 (Figure 1), which was previously demonstrated to bind pro-apoptotic siRNAs providing efficient uptake of complexes by HeLa cells. Importantly, the siRNA binding by AE2G3 dendrimers is reversible, and complexes are able to dissociate spontaneously upon long incubation [19]. siRNA-dendrimer polyelectrolyte complexes (dendriplexes) induced apoptosis in tumor cells that resulted in the sharp decrease of viability [9]. Importantly, the dendrimer kept its carrier functionality in the presence of proteins in a culture medium. Our work is supposed to be the first step of the proof-of-concept study of the dendrimer-based approach for the delivery of therapeutic nucleic acids into cancer stem cells, key actors of tumor development and resistance.

We have tested the effects of AE2G3/siLyn dendriplexes on 2 cell GSC cultures: JHH520, which is known to express Lyn kinase, and NCH644, which was stated as having low or absent Lyn expression: the expression of the cell lines was analyzed using the data set GSE181315 [2]. Interestingly, it contradicts previous data mentioning the quite low Lyn expression in GSCs derived from patient samples [49]. Flow cytometry assays confirmed the efficient accumulation of fluorescent-labelled dendriplexes in tumor cells (Figure 5), despite their relatively large size. We suggest that pre-formed dendriplexes are reorganized upon incubation in cell media during transfection. After 4 h of exposure to AE2G3/siLyn dendriplexes bearing fluorescently labelled siRNA, ca. 80% of cells were transfected. It is worth noting that, when using the standard transfecting agent Lipofectamine as transfection agent in comparator studies, only ca. 15% of BTSC233 and ca. 20% JHH520 cells could be transfected.

JHH520 cells demonstrated higher sensitivity to siLyn-containing treatment (free RNA or complexes with AE2G3 and Lipofectamine 3000), and the dendriplexes had slightly lower toxicity in comparison with siLyn or AE2G3 taken alone, but higher compared to Lipofectamine 3000/siLyn. NCH644 cells were sensitive to AE2G3 treatment, but not to siLyn and siLyn-complexes. Interestingly, the same effects were observed upon treating cells with dasatinib, a specific kinase inhibitor. These findings further support the role of Lyn in promoting the proliferation of glioblastoma cells.

JHH520 cells were more sensitive to dasatinib or free siLyn treatment, in comparison with “Lyn-negative” NCH644 cells. As JHH520 models a mesenchymal glioblastoma, and although a comparison is made between two lines only, this data is supportive of recent reports that patients suffering from the mesenchymal glioblastoma subtype might benefit most from receiving dasatinib therapy [50]. Verification studies to assess the target septicity by using the genetic enforced suppression of Lyn in Lyn-positive cell models and comparison to phenotypes of isogenic controlled conditions under therapy exposure are warranted to make any statement on the adversity risk of the developed platform. Moreover, in vivo application of the dendriplexes needs to prove the systemic tolerability of the nanodrug in physiological acceptable concentrations.

We have also attempted to follow the changes in the Lyn protein level upon treatment with siRNA. However, Lyn appeared to be quite poorly visualized due to its low abundance, even in JHH520 cells. Therefore, Western blot assay permitted the visualization of the modulation of Lyn expression in cells treated with dendriplexes but could not provide statistically significant data on the protein downregulation (Figure S2).

It is worth noting that the secretion of IL-10 is not significantly changed upon treatment with AE2G3 (with a tendency to decrease) or AE2G3/siLyn and increased upon Lipofectamine 3000 treatment. IL-10 is an immunosuppressive factor which promotes the shift of microenvironment cells to the tumorigenic phenotype and decreases the immune response [51].

Thus, we demonstrated that our formulations transport RNAi efficiently into the tumor cells, but the integral activity of the drug is rather low. Despite the clinical attractiveness of Lyn kinase as a target, it is likely not optimal, being the protein of relatively low or unstable expression. In our previous work, we have observed the pronounced cytotoxic effects of pro-apoptotic siRNAs at a concentration 25 nM [9]; however, in GSCs, the effects were observable only at a siLyn concentration of 100 nM.

Along with the use as a vehicle for therapeutic nucleic acid delivery, phosphorus dendrimers can be also used as an antitumor agent per se, as mentioned above. Despite the common consideration that a drug vehicle should be non-toxic, we can state that in the case of antitumor therapy, certain cytotoxic activity of a carrier could bring some synergy to the treatment by targeting alternative cell death pathways in comparison with a cargo. We have studied the effects of AE2G3 dendrimer on tumor cells, with the variety of cell lines in use having been expanded (Figure 2). On this step we only studied tumor models, but the wider screening of dendrimer toxicity on healthy cells will be necessary in the future.

Dendrimer was found to have dose-dependent toxic effect. To separate cytotoxic effects of dendrimer itself and dendrimer-siRNA complexes, we have run control experiments using dendrimer alone at the same concentrations as in complexes, and siRNA delivered by Lipofectamine. Importantly, the toxicity of the dendrimer at the concentrations used (up to 830 nM) did not differ significantly from the non-treated control.

Interestingly, at high doses of the dendrimer, the increasing of cell viability could be observed. It could be related to the aggregation of dendrimer molecules or the co-aggregation of dendrimers with cell medium components at higher concentrations, with these aggregates being less toxic to cells. In general, dendrimers demonstrated higher toxicity on tumor cells compared to temozolomide. These facts taken together allow us to suppose that the cationic phosphorus dendrimer AE2G3 could be used as a therapeutic agent in resistant tumors.

On the next step, we studied whether the treatment could modulate the expression of immune checkpoint molecules on tumor cells surface, since those are in the center of ongoing discussions on improving the care of glioblastoma patients with immune therapy. The PD-L1 ligand interacts with a PD-1 receptor on the group of immunocompetent cells, providing a reduced antitumor immune response; the PD-1/PD-L1 axis is actively involved in the promotion of glioblastoma cells invasion [52,53]. Previous studies found correlating patterns of Lyn and PD-L1 expression; hypothetically, Lyn expression is supposed to be a predictor of the efficacy of anti-PD-1 and anti-CTLA-4 immunotherapy [47]. TIM-3 is a T cell immunoglobulin domain and mucin domain 3 negative checkpoint molecule. It is expressed on glioma cells predicting worse survival, and its role seems to be similar to PD-L1 function [54,55]. Moreover, TIM-3 acts in Lyn-related signaling pathways [56]. CD47 is a transmembrane glycoprotein, acting as a “do not eat me” antiphagocytic signal [57]; its high expression is a negative prognostic marker in gliomas, and the inhibition of CD47 in different cancer models suppresses tumor growth [58]. TIM3 and CD47 are highly expressed on tumor stem cells and could be used as markers for appreciating the maintenance of this cell population under exogenous stress [54,58]. We have found that treatment with the AE2G3 dendrimer significantly increases the expression of TIM3 and PD-L1 on the cell surface in comparison with control, with no such effect being observed for temozolomide and dasatinib. These effects were identical in three cell lines. Unlike TIM3 and PD-L1, CD47 expression has been shown to vary in different cell lines, and responses to the treatment were also different. In the BTSC233 line, no significant differences were found between CD47 expression in control cells and cells treated with dendrimer, temozolomide and dasatinib. In JHH520 cells, both dendrimer and dasatinib reduced the expression of CD47 in comparison with control, and no significant effect was observed for temozolomide. In NCH644, the dendrimer increased the expression of CD47, and no effect was observed for temozolomide and dasatinib. These functional changes and their effect on interactions between the tumor and its microenvironment deserve further elucidation. Possible explanations could also be related to the metabolic state of tumor cells: it was found recently that some types of antitumor therapy could lead to increased PD-L1 expression on tumor cells and bone marrow stromal cells [59] which could probably be the marker of metabolic stress [60,61]. This phenomenon potentially can be investigated for therapeutic protocols including AE2G3 and checkpoint inhibitors which are used, for example, for the anti-PD-1/PD-L1 therapy.

**Figure 6:**
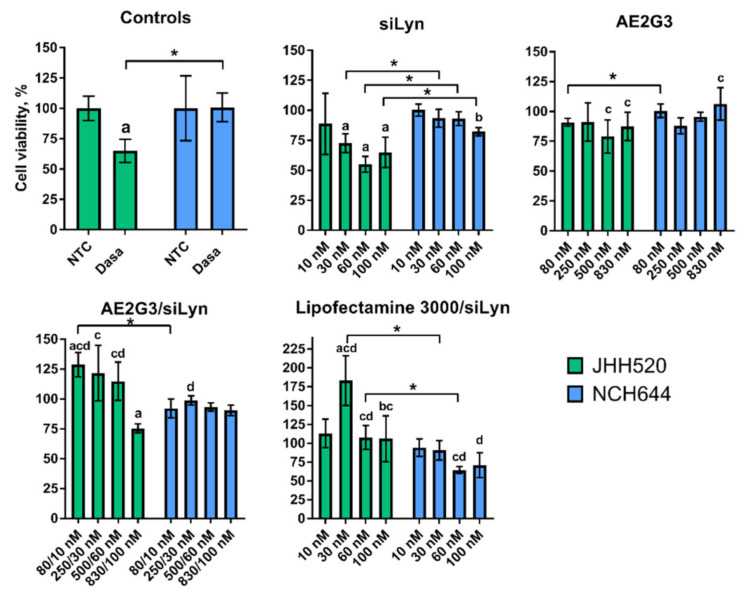
Evaluation of JHH520 and NCH644 cell (both—GSCs) viability after treatment by AE2G3/siLyn dendriplexes (AE2G3 and siLyn concentrations marked according to the charge ration) in comparison with non-treated control (NTC), dasatinib (Dasa, 100 nM), free siLyn (10 nM, 30 nM, 60 nM, 100 nM), free AE2G3 (80 nM, 250 nM, 500 nM, 830 nM) and complexes of siLyn with Lipofectamine 3000 (Lipo). “a” marks significant difference in comparison with NTC, “b” marks significant difference in comparison with dasatinib, “c” marks significant difference in comparison with free siLyn, and “d” marks significant difference in comparison with free AE2G3. Bars with asterisk (*) mark significant differences between cell lines.

The fact that glioblastoma stem cells are particularly resistant to TMZ is generally acknowledged as contributing to the unsatisfying clinical success of the current standard of care. Given its recognition as clinical consensus, the effects observed with this drug serve as the benchmark for new interventions, such as in our project, where our comparator arm TMZ is widely used. Importantly, the use of dasatinib exhibits better results in suppressing the viability of tumor stem cells expressing Lyn (see Figure 6).

## 5. Conclusions

Herein, we have designed antitumor nanoformulations for molecular-targeted therapy based on siRNA and polycationic phosphorus dendrimers acting as a toolbox for the development of efficient therapeutics against tumor stem cells. Although further studies are required to elucidate the fine effects of such nanoformulations such as off-target adversity risk and in vivo bioavailability at the target site when systemically applied into xenograft bearing animal models application, the present proof-of-concept study provides evidence to validate our dendrimer-based nanoformulations for anti-glioblastoma therapy. Elevated Lyn mRNA expression levels in glioma tissue possess significant negative clinical predictive value, promoting its potential as a therapeutic target for future molecular-targeted treatments.

## Data Availability

Data supporting the conclusions of this article will be made available by the authors upon reasonable request.

## Supplementary Materials

The following supporting information can be downloaded at: https://www.mdpi.com/article/10.3390/ijms23105691/s1.
