# Supplementary material for: In Vitro Validation of the Therapeutic Potential of Dendrimer-Based Nanoformulations against Tumor Stem Cells
Source: Int J Mol Sci. 2022 May 19;23(10):5691. doi: 10.3390/ijms23105691 (PMC9143703; doi:10.3390/ijms23105691)
Supplement: Supplementary file 1 [file ijms-23-05691-s001.zip › ijms-1696389-supplementary.pdf]

International Journal of *Molecular Sciences*

Supplementary Materials: *In vitro* validation of the therapeutic potential of dendrimer-based nanoformulations against tumor stem cells

Nadezhda Knauer, Valeria Arkhipova, Guanzhang Li, Michael Hewera, Ekaterina Pashkina, Phuong-Hien Nguyen, Maria Meschaninova, Vladimir Kozlov, Wei Zhang, Roland S Croner, Anne-Marie Caminade, Jean-Pierre Majoral, Evgeny K. Apartsin, Ulf Dietrich Kahlert

AE2G3 dendrimer efficiently protects siRNA from digestion with RNAse A

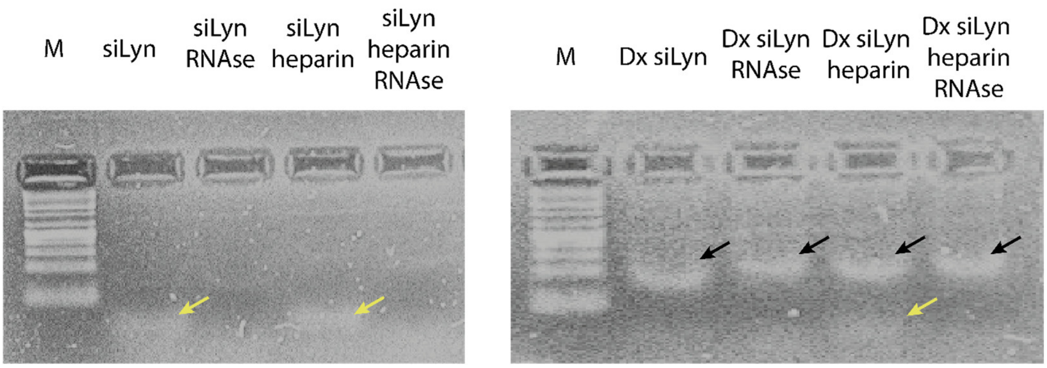

Figure S1. Evaluating of protective properties of AE2G3 on RNA upon RNase treatment (mobility in 3% agarose gel). Yellow arrows mark the RNA bands, black arrows mark the siLyn-dendrimer complexes.

Western blot assay permits to visualize the modulation of Lyn expression in cells treated with dendriplexes but cannot provide statistically significant data on the protein downregulation

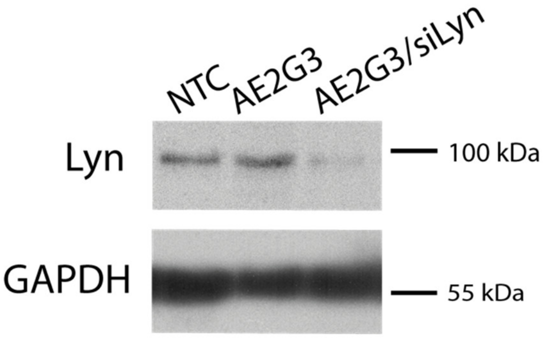

Figure S2. Representative Western blot image of Lyn and GAPDH proteins' levels in JHH520 cells before and after treatment with dendrimer-siLyn complexes.

Free AE2G3 does not change IL-10 secretion by NCH644 cells

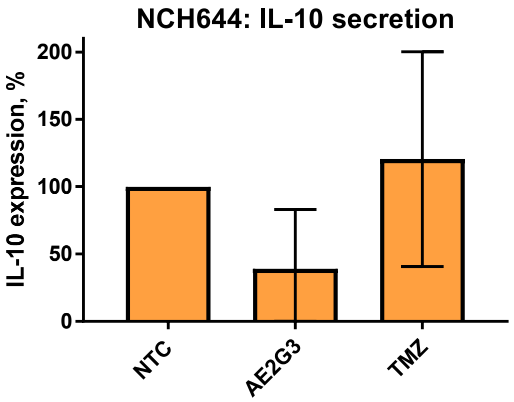

Figure S3. Evaluation of IL-10 secretion by NCH644 after treatment by free molecules. Data shown as percent on non-treated control (NTC) values.

We did not find any significant changes of IL-10 secretion after 72 hours of dendrimer or TMZ treatment.

Dendrimer/siRNA dendriplexes do not significantly change IL-10 by JHH520 tumor cells

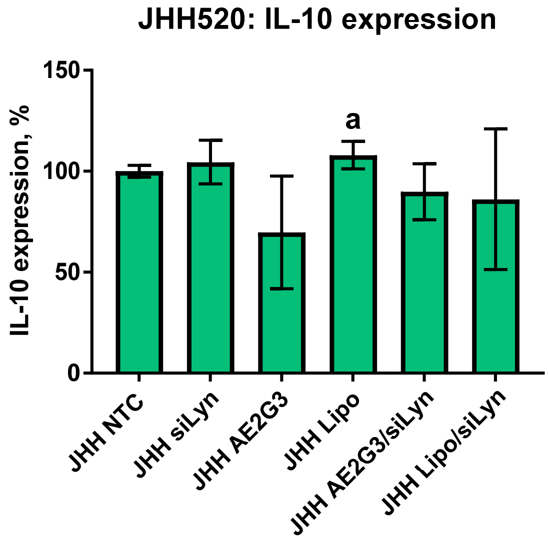

Figure S4. Evaluation of IL-10 secretion by JHH520 after treatment. Data shown as percent on non-treated control (NTC) values. Lipofectamine (Lipo) was used as a standard carrier. The letter a marks significant difference ($p<0,05$) with NTC.

We did not find any significant changes of IL-10 secretion after dendrimer or dendriplexes treatment, but Lipofectamine 3000 treatment slightly increased this parameter in comparison with NTC.
